# Quality assurance of 3D-printed patient specific anatomical models: a systematic review

**DOI:** 10.1186/s41205-024-00210-5

**Published:** 2024-03-27

**Authors:** Martin Schulze, Lukas Juergensen, Robert Rischen, Max Toennemann, Gregor Reischle, Jan Puetzler, Georg Gosheger, Julian Hasselmann

**Affiliations:** 1 Department of General Orthopedics and Tumor Orthopedics, University Hospital Muenster, 48149 Münster, Germany; 2 Clinic for Radiology, University Hospital Muenster, 48149 Muenster, Germany; 3 Qualified AM GmbH, 82407 Wielenbach, Germany; 4 Department of Mechanical Engineering, Materials Engineering Laboratory, University of Applied Sciences Muenster, 48565 Steinfurt, Germany

**Keywords:** Anatomical models, Quality assurance, Medical additive manufacturing, 3D-printing, Segmentation error, Digital editing error, Printing error, Error classification

## Abstract

**Background:**

The responsible use of 3D-printing in medicine includes a context-based quality assurance. Considerable literature has been published in this field, yet the quality of assessment varies widely. The limited discriminatory power of some assessment methods challenges the comparison of results. The total error for patient specific anatomical models comprises relevant partial errors of the production process: *segmentation error (SegE)*, *digital editing error (DEE)*, *printing error (PrE)*. The present review provides an overview to improve the general understanding of the process specific errors, quantitative analysis, and standardized terminology.

**Methods:**

This review focuses on literature on quality assurance of patient-specific anatomical models in terms of geometric accuracy published before December 4th, 2022 (*n* = 139). In an attempt to organize the literature, the publications are assigned to comparable categories and the *absolute values of the maximum mean deviation (AMMD)* per publication are determined therein.

**Results:**

The three major examined types of *original structures* are teeth or jaw (*n* = 52), skull bones without jaw (*n* = 17) and heart with coronary arteries (*n* = 16). VPP (vat photopolymerization) is the most frequently employed basic 3D-printing technology (*n* = 112 experiments). The median values of *AMMD* (AMMD: The metric AMMD is defined as the largest linear deviation, based on an average value from at least two individual measurements.) are 0.8 mm for the *SegE*, 0.26 mm for the *PrE* and 0.825 mm for the total error. No average values are found for the *DEE*.

**Conclusion:**

The *total error* is not significantly higher than the *partial errors* which may compensate each other. Consequently *SegE, DEE* and *PrE* should be analyzed individually to describe the result quality as their sum according to rules of error propagation. Current methods for quality assurance of the segmentation are often either realistic and accurate or resource efficient. Future research should focus on implementing models for cost effective evaluations with high accuracy and realism. Our system of categorization may be enhancing the understanding of the overall process and a valuable contribution to the structural design and reporting of future experiments. It can be used to educate specialists for risk assessment and process validation within the additive manufacturing industry.

**Graphical Abstract:**

Context of the figures in this review. Center: Fig. 5+ 7; top (blue): Fig. 8; right (green): Fig. 9; bottom (yellow): Fig. 10; left (red): Fig. 11. A version in high resolution can be found online in the supplementary material.

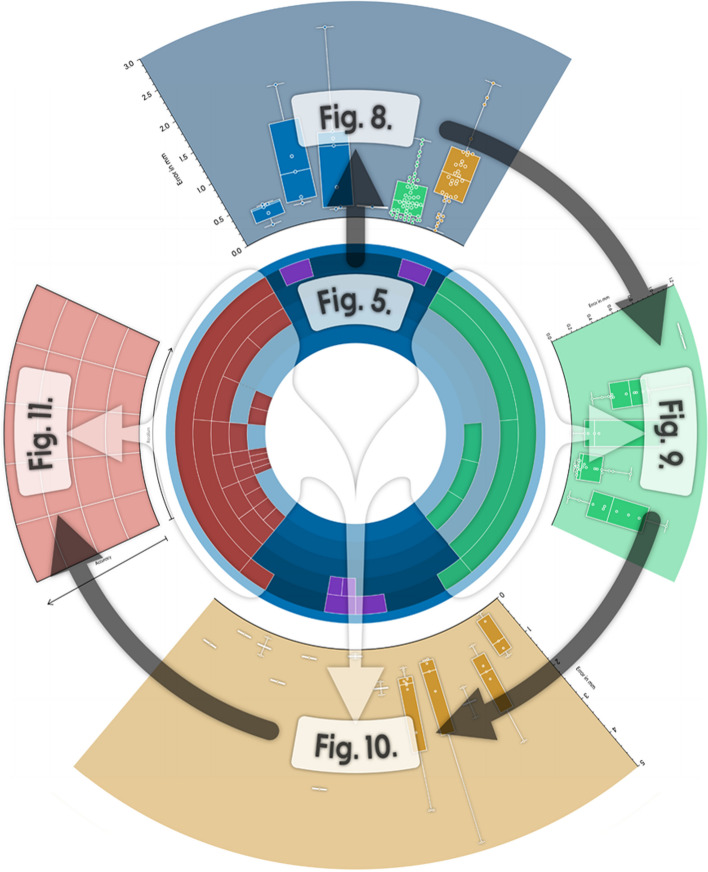

**Supplementary Information:**

The online version contains supplementary material available at 10.1186/s41205-024-00210-5.

## Background

### 3D-printing in medicine

The technologies of 3D-printing in medicine have revolutionized patient care and treatment strategies. From fabricating patient-specific anatomical models [1, 2] to producing customized surgical guides [3, 4] and implants [5, 6], 3D-printing offers rapid, precise and tailored solutions that facilitate medical education [7, 8], improve patient information [9, 10] and enhance procedural and surgical outcomes [11, 12].

In the responsible use of 3D-printing in medicine, sufficient and context-based quality assurance must be guaranteed in terms of patient safety. This implies the need for a quality assurance program covering the entire production process and interfaces to adjacent processes. This essentially includes manufacturing tolerances, which certainly may vary depending on the subsequent use case of the 3D-print. Determining these can be done in a variety of ways and is not trivial. For a comparison with defined manufacturing tolerances, the error must be accurately assessed.

### Quality assurance of 3D-printed patient specific anatomical models

The 3D Printing Special Interest Group of the Radiological Society of North America has already established guidelines for medical 3D-printing, which describe recommendations for quality assurance [13]. Although considerable literature has been published in this field, the methods used to assess quality vary widely. Due to the limited discriminatory power of some assessment methods the comparison of results between different authors is challenging. This aspect has already been discussed by Illi et al. [14] and Chae et al. [15].

The present review focuses on the literature on quality assurance of patient-specific anatomical models in terms of geometric accuracy published before December 4th, 2022. As an attempt to organize the literature, the included publications are assigned to comparable categories and the *absolute values of the maximum mean deviation* (AMMD) per publication are determined therein. The present review provides an overview from the perspective of the clinical user to improve the general understanding of the process specific errors. The goal is to facilitate access for future systematic approaches and reporting through suggested standardized terminology.

### The medical 3D-printing process and its errors

The production of patient-specific anatomical models is a multi-step process, in which each individual step may involve a partial error.

**Fig. 1:**
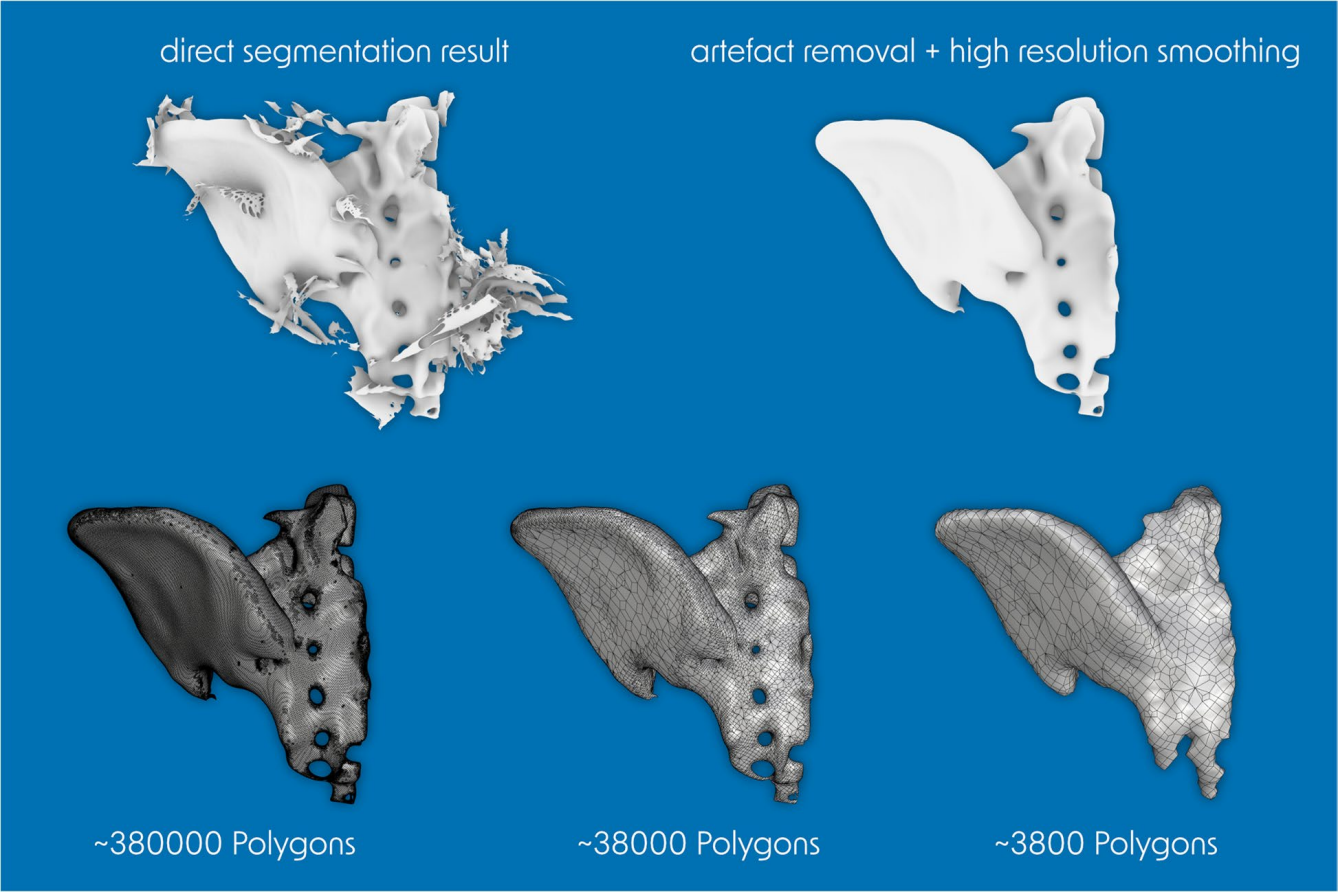
Visualization of artefact removal, smoothing and reduction of mesh density with a loss of anatomical details. A left hemipelvis is shown as an example. When the number of polygons is reduced by a factor of 100, the anatomical details of the neuroforamina and sacral segments are lost

The production process begins with imaging of the original structure, e.g. computed tomography from patients (clinical setting), cadaveric specimens, anatomical models or phantoms (experimental setting). The resulting data sets are stored in Digital Imaging and Communications in Medicine (DICOM) file format. A virtual three-dimensional model is generated from the DICOM data sets by segmentation. In most cases the standard tessellation language (STL) file format is used for saving a model. This format is a translation of the model into a three-dimensional mesh structure of triangles and normal vectors. These STLs are prone to errors such as artefacts, mesh gaps and misorientation of the normal vectors, but can be corrected by various, partly automated, partly manual repair methods including smoothing (digital editing). However, if the resolution of the mesh is low, the model may deviate significantly from the original (Fig. 1).

Digital editing techniques help to generate a print-STL file of good quality, which is translated into a machine-readable code (slicing). This code, which may be printer-specific, is then interpreted by the 3D-printer in order to build a 3D object layer by layer. After the built is completed, a material and printing technology-dependent post-processing is necessary, e.g. removal of support structures or a curing with ultraviolet light.

**Fig. 2:**
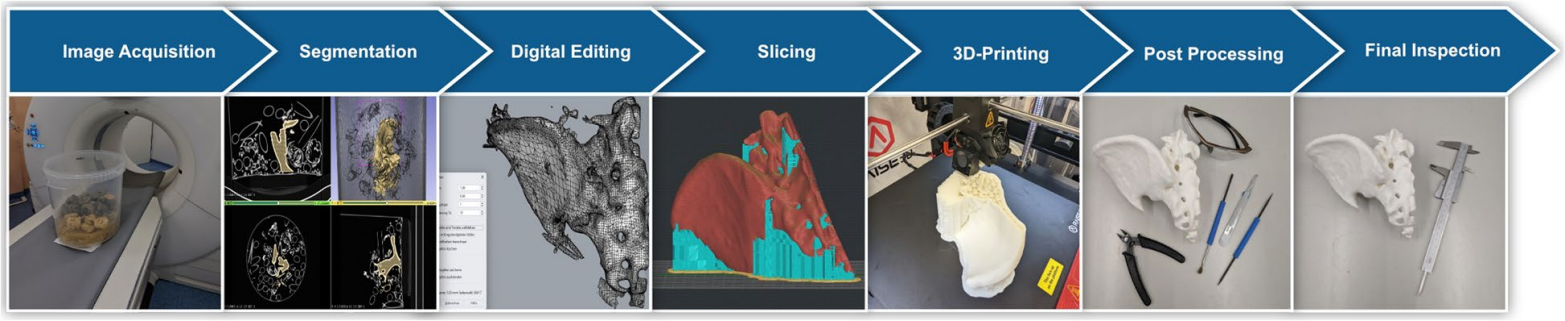
Medical 3D-printing process for the production of patient specific anatomical models

Figure 2 shows the process for the production of patient-specific 3D-printed anatomical models.

**Fig. 3:**
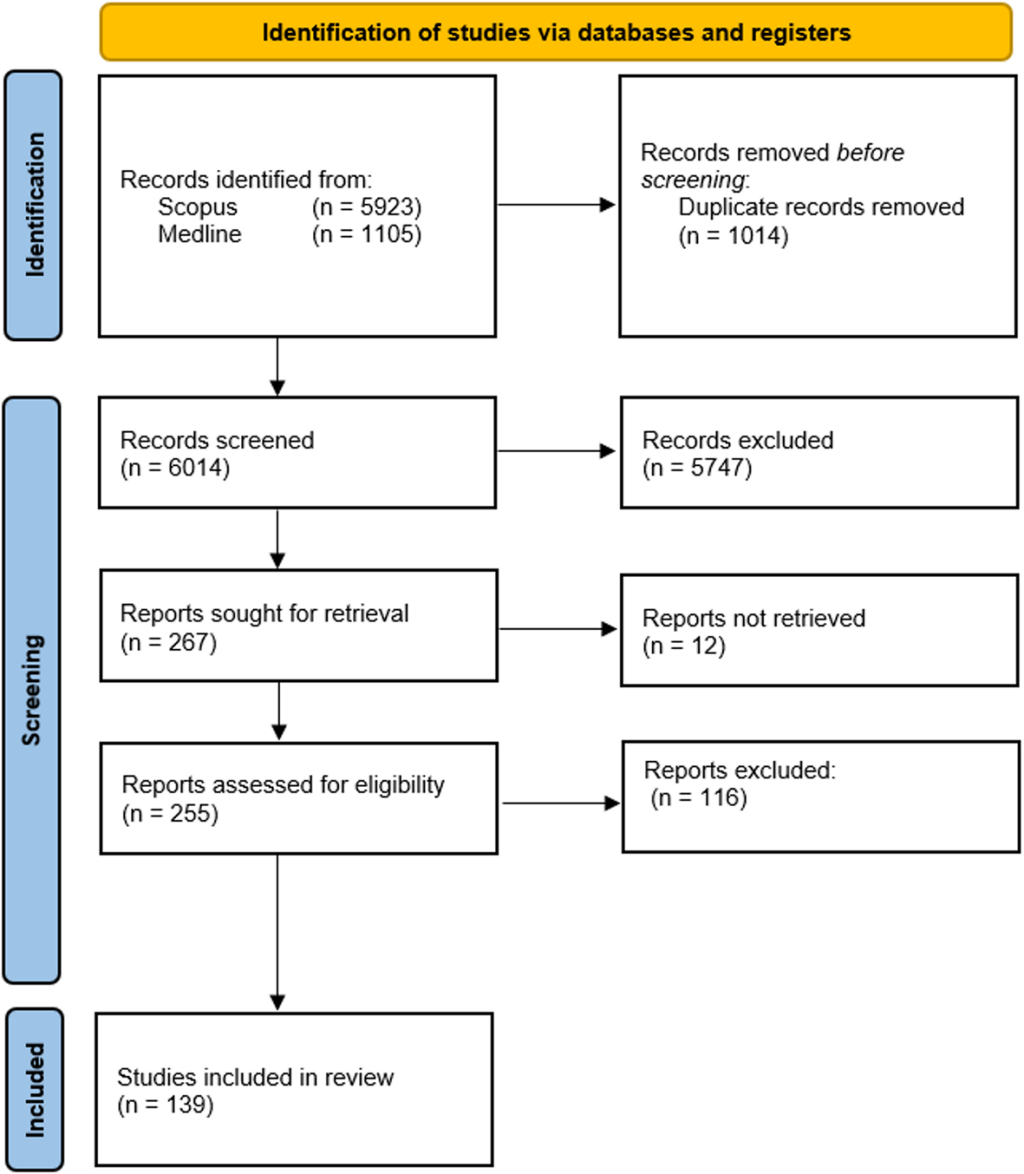
PRISMA flow chart resulting in 139 articles included [66]

The ISO (International Organization for Standardization) 5725–2:2019 standard describes the accuracy of measurement methods in terms of *trueness* and *precision* [16]. In medical 3D-printing, those can be determined for each individual process step. *Trueness* is determined by calculating the error between the reference and the result of a single sub-step within the overall process. *Precision* is assessed by comparing multiple results of a single sub-step of the overall process when performed repeatedly [17, 18].

## Materials and methods

### Screening of the literature

A systematic literature search was conducted in Scopus and PubMed (Medline database) according to the Preferred Reporting Items for Systematic Reviews and Meta-Analyses (PRISMA) guidelines. The following search algorithms were used.

**Scopus:** TITLE-ABS-KEY ((accuracy OR (“quality control”) OR (“quality assurance”) OR assessment) AND ((models OR modeling) AND (“3D printing” OR “3D printed” OR “additive manufacturing”)) AND NOT bioprinting).

**Medline:** (accuracy[Title/Abstract] OR (“quality control”[Title/Abstract]) OR (“quality assurance”[Title/Abstract]) OR assessment [Title/Abstract]) AND ((models[Title/Abstract] OR modeling[Title/Abstract]) AND (“3D printing”[Title/Abstract] OR “3D printed”[Title/Abstract] OR additive manufacturing [Title/Abstract])) NOT bioprinting [Title/Abstract].

The initial search on December 4th 2022 resulted in 5923 search results on Scopus and 1105 on PubMed, respectively. The results were exported using research information system format (RIS) and imported into Citavi (Swiss Academic Software GmbH, V6.1, Wädenswil, Switzerland) for deduplication. 1014 duplicate records in total were removed before screening. 6014 records were screened by title of which 5747 were excluded manually due to exclusion criteria as shown in Table 1.

**Table 1:** In- and exclusion criteria for title screening

Inclusion	Example	Exclusion	Example
Criteria		Criteria	
quality assessment of medical models	[19]	clinical studies	[20]
tolerances of additive manufactured objects for clinical use	[21]	no medical context	[22]
additive manufactured models in medicine	[23]	evaluation of material properties	[24]
improving accuracy of 3D-printing	[25]	usage of printed models for surgery simulation instead of cadavers	[26]
review 3D-printing in medicine	[27]	mechanical properties of parts produced with metal powder bed fusion	[28]
quality assessment of segmentation	[29]	effect on patient education	[30]
comparison of different printing processes	[31]	assessment of using 3D-printing for education of professionals	[8]
quality assessment of printing process	[32]	assessment of surgery results	[33]
review and validation	[14]	using 3D-printed models as phantoms for radiology, nuclear medicine or radio therapy	[34]
		using 3D-printing for mechanical analyses	[35]
		accuracy assessment of surgical guides	[36]
		general opportunities of 3D-printing in medicine	[37]
		planning and simulation of surgeries using 3D-printing	[38]
		analysis and prediction of printing quality from the perspective of engineers	[39]
		functional models	[40]
		analysis of energy consumption or cost	[41]
		biomedical implants	[42]
		only review	[43]
		general improvements of printing process from the perspective of engineers	[44]
		models that simulate haptic reality	[45]
		assessment of color	[46]
		review of 3D-printing for surgical teaching	[47]

Of the remaining 267 reports 12 were not retrieved (three because full texts were not accessible and nine because language was not English or German). Out of the remaining 255 reports, 116 were excluded after full text screening due to exclusion criteria as shown in Table 2. 139 Studies were included in this review **(**Fig. 3)

**Table 2:** Exclusion criteria for full text screening with one example for each

Exclusion	Example
Criteria	
influences on the printing process from the engineers’ perspective	[32]
influences of slicing tools on printing accuracy from the engineers’ perspective	[48]
accuracy of implants	[49]
assessment of special post processing techniques like vapor smoothing	[50]
assessment of surface properties	[51]
assessment of STL-export from CAD software	[52]
evaluation of ageing process of printed models	[53]
evaluation of thermoformed appliances	[54]
using 3D-printed models for assessment of image acquisition	[55]
phantoms for MRI	[56]
evaluation of an experimental full-automatic segmentation algorithm	[57]
evaluation of 3D-printed models as diagnostic tool in comparison to the standard	[58]
accuracy assessment of 3D-printed surgical guides	[59]
realistic surgery models for procedure assessment	[60]
evaluation of 3D-printed models as a diagnostic tool in comparison to the standard	[58]
only visual evaluation	[61]
only review	[62]
only clinical study	[63]
only case report	[64]
no validation	[65]

### Categories for classifying measurements

When quantifying errors in the medical 3D-printing processes, it is important to consider which steps of the whole process are evaluated and how this evaluation takes place. One example for this is that the accuracy of the segmentation process is greatly influenced by whether adjacent tissue (simulated or real) is included in the image acquisition, or whether the *original structure* is scanned in air [67]. The following overview presents categories which contain comparable measurements from the literature.

#### Individual and combined errors in medical 3D-printing processes

**Fig. 4:**
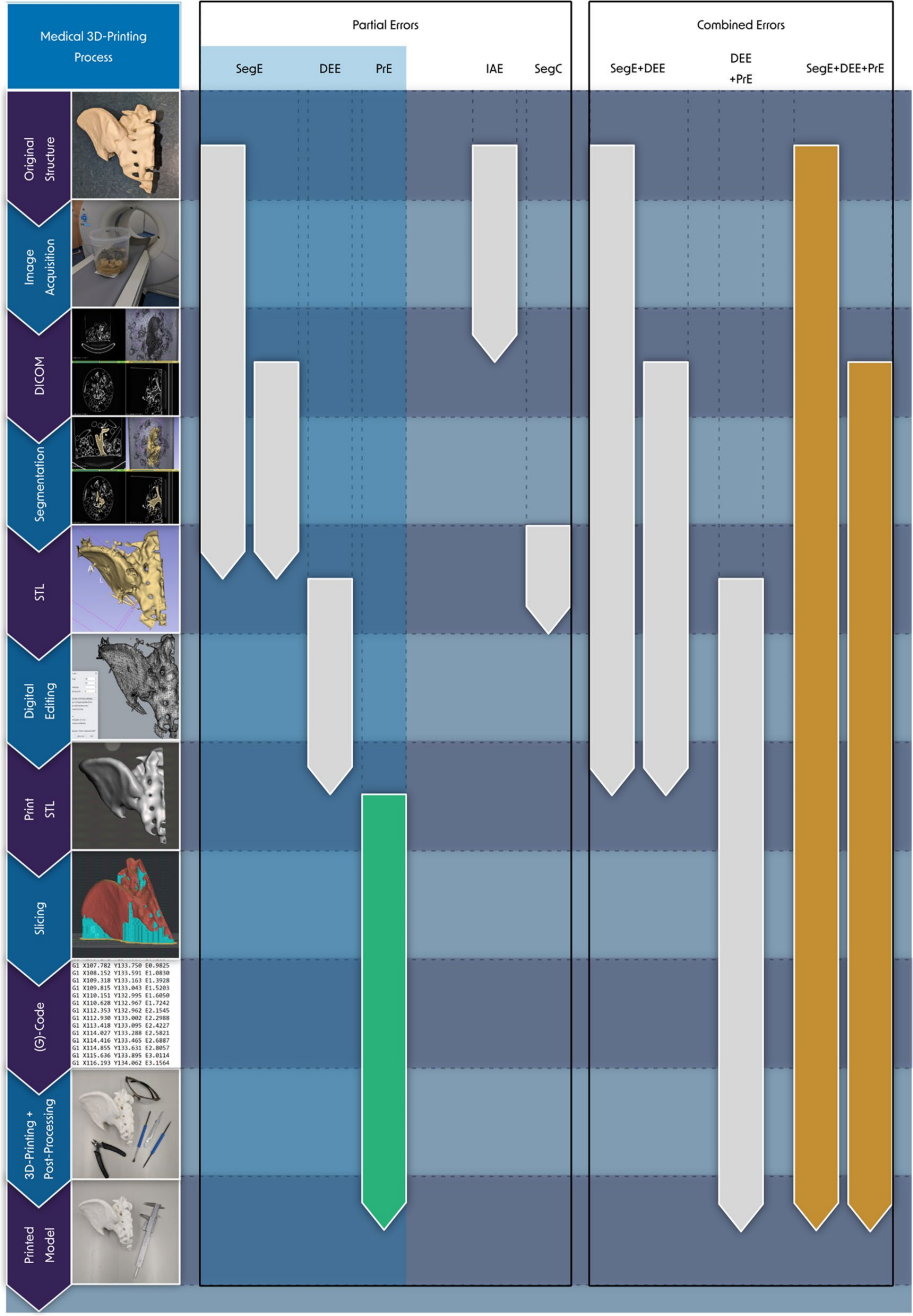
Medical 3D-printing process for the production of patient specific anatomical models and its errors. In addition to the steps of the process shown in Fig. 2, the intermediate results are also shown. The arrows indicate which of the intermediate process results are compared to determine the respective main types of error, including the differentiation of *combined* and *partial errors*. Highlighted with blue box: Errors that should be evaluated individually according to the guidelines of the RSNA for medical 3D-printing [13]. SegE: *Segmentation Error*, DEE: *Digital Editing Error*, PrE: *Printing Error*, IAE: *Image Acquisition Error*, SegC: *Segmentation Comparison Error*. PrE: most frequently evaluated *partial error*, SegE+DEE + PrE: most frequently evaluated *combined error*

Figure 4 shows the process for the production of patient-specific 3D-printed anatomical models, including the main types of errors that can occur. In addition to the process shown in Fig. 2, the intermediate results of each sub-step are also shown. Partial errors and their possible combinations are distinguished. The combined investigation of partial errors is possible if they refer to directly consecutive process steps. Thus, the combination of segmentation error (SegE) and digital editing error (DEE) can be examined as well as the combination of DEE and and printing error (PrE). The total error can be examined as a combination of SegE, DEE and PrE.

As *image acquisition* not only represents the initial step of the production process but also serves as a tool for measuring the *original structure*, a peculiarity arises for errors involving the SegE: there are two possible process steps where reference measurements can be taken: a) directly on the original structure e.g. using a caliper or b) on the DICOM data using linear measurement tools. Assuming that *the image acquisition error* (IAE) is an externally controlled parameter to our model with tight error tolerances guaranteed by the specialized discipline of medical physics according to manufacturer specifications [68], in this publication, the term “*segmentation error*” refers not only to the individual *segmentation error* (SegE), but also to the combined error of *image acquisition* (IAE) and *segmentation* (IAE + SegE). Consequently, the individual IAE is not separately considered in the combined error analyses beyond the SegE.

In general, the focus is on the *trueness* of various steps involved in the process of creating patient-specific anatomical models. However, regarding the *segmentation error*, publications that evaluate *precision* are also included, as this step requires the most manual input.

**Fig. 5:**
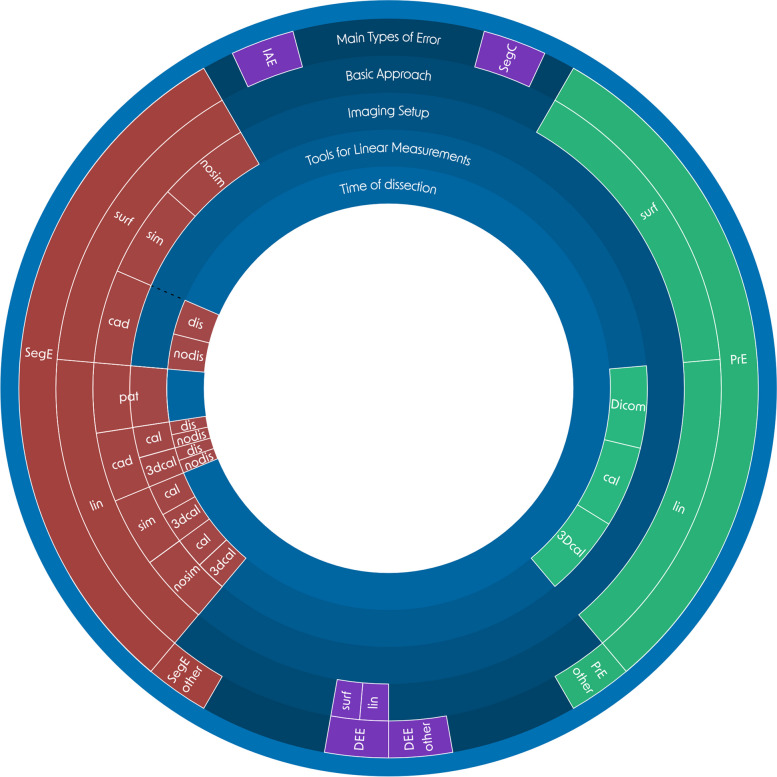
Visualization of the subcategories for the detailed analysis of applied methods. *Main types of error*: IAE: *Image Acquisition Error*, SegE: *Segmentation Error*, SegC: *Segmentation Comparison Error*, DEE: *Digital Editing Error*, PrE: *Printing Error*; basic approach: linear (lin) or surface (surf) deviation based analysis; imaging setup: experimental setups of the image acqusition: nosim: artifical model and no simulation of adjacent tissue, sim: artifical model and simulation of adjacent tissue, cad: cadaver study, pat: scan of patients due to clinical questions; tools for linear measurements: DICOM: CT-Scan of 3D-printed model and linear measurements on resulting DICOM data, cal: caliper, 3Dcal: 3D-scan and virtual linear measurements, pat: linear measurements on patient DICOM data; time of dissection (for cadaver studies): dissection is done before image acquisition (dis) or after image acquisition (nodis); could not be assigned to any of the introduced categories (other). A detailed description for each subcategory can be found in Appendix A

In the following section, the main types of errors are defined and the methodology for categorizing experiments to evaluate medical 3D-printing processes is outlined. The categorization of the included literature is based on the methods for conducting measurements on both the *original structures* and the *printed models*, as well as the experimental setup of the *image acquisition*. A visualization of the error subcategories is shown in Fig. 5 and a more detailed description of all subcategories can be found in Appendix A.

#### Evaluation of the *segmentation error* (SegE)

The SegE is defined as the deviation between the *original structure* and the direct result of the segmentation process. All experiments assessing SegE are further subdivided by the measurement methods used and the experimental setups of the image acquisition.

#### Evaluation of the *digital editing error* (DEE)

The DEE is defined as the deviation between the direct result of the segmentation process and the *print-STL*. All experiments assessing the DEE are further subdivided by whether a linear measurement-based or a surface deviation-based analysis was performed.

#### Evaluation of the *printing error* (PrE)

The PrE is defined as the deviation between the *print-STL* and the *printed model*. All experiments assessing the PrE are further subdivided by the measurement tools used.

#### Evaluation of the *image acquisition error* (IAE)

The IAE is defined as the deviation between the *original structure* and the result of the *image acquisition* (DICOM data set).

#### Evaluation of the *segmentation comparison error* (SegC)

The SegC is defined as the precision of the segmentation process when it is performed repeatedly, e.g. by different users or with different software.

#### Combination of *segmentation error* and *digital editing error* (SegE+DEE)

The combination of SegE and DEE is defined as the deviation between the *original structure* and the *print-STL*. All experiments assessing the combined error of SegE and DEE are further subdivided by measurement methods and experimental setups of the *image acquisition*. For this purpose, the SegE subcategories are used.

#### Combination of *digital editing error* and *printing error* (DEE + PrE)

The combination of DEE and PrE is defined as the deviation between the direct result of the segmentation process and the *printed model*. All experiments assessing the combination of DEE and PrE are further subdivided by the measurement tools used. For this purpose, the PrE subcategories are used.

#### Combination of *segmentation error, digital editing error* and *printing error* (SegE+DEE + PrE)

The combination of SegE, DEE and PrE is defined as the deviation between the *original structure* and the *printed model*. All experiments assessing the *total error* are further subdivided by measurement methods and experimental setups of the *image acquisition*. For this purpose, combinations of SegE and PrE subcategories are used.

#### Subcategories for the detailed analysis of applied methods

Sections 2.2.2 to 2.2.9 defined the main error types describing which process steps of the medical 3D printing process are compared with each other. However, it should also be considered which methods are applied for this comparison, as they can differ significantly in their realism and accuracy. To describe the applied methods in detail, they were analyzed on four levels: 1. the basic approach, 2. the imaging setup, 3. the tools for linear measurements, and 4. the time of the dissection. Within the basic approach, a distinction is made between methods that apply linear measurements (lin), e.g. between two landmarks, and those that apply software-supported surface comparisons (surf). In the case of surface comparisons, for example, the resulting digital model from an optical 3D scan of an original structure could be compared with the direct segmentation result after an iterative alignment. The imaging setup, together with the timing of the dissection, is primarily responsible for the realism of an experiment. A distinction was made between artificial models scanned in air environment (nosim), artificial models scanned with simulation of adjacent tissue (sim), cadaver studies (cad) and real patients (pat). For linear measurement tools, calipers (cal), virtual software-supported calipers after a 3D scan was conducted (3Dcal), and CT combined with linear measurements on resulting DICOM data for measurements of printed models (DICOM) were distinguished. For cadaver studies, the realism of an experiment also depends on whether the dissection was done before imaging (dis) (e.g., scanning dry bones) or after imaging (nodis) (e.g., scanning wet specimens). Figure 5 shows a visualization of the subcategories for the main types of error, including the four levels for method analysis “basic approach”, “imaging setup”, “tools for linear measurements” and “time of dissection”.

### Descriptive analysis

The 3D-printing technology used is reported for all publications that evaluate *printing errors*. The proprietary technologies of manufacturers are categorized into the three main groups of the basic 3D-printing technologies: “curing of liquid photopolymers”, “extrusion of tough masses through nozzle” and “melting/sintering/binding of powder” (Table 3).

**Table 3:** Basic 3D-printing technologies, printing technologies as named by manufacturer of device and description for each of them

Basic technology	Printing technology as described in publication or by manufacturer	Description
curing of liquid photopolymers	Multi jet printing (MJP) / Polyjet printing(PJP)	A photocurable polymer is released by a printhead and is directly cured afterwards by UV light.
	Digital light processing (DLP)	A build plate inside a tank filled with liquid resin is exposed with UV light from underneath. Each exposure adds a whole layer to the built plate. The buid plate moves upwards after every light exposure.
	Stereolithography (SLA)/Scan, Spin and Selectively Photocuring	Mirrors redirect a laser beam through a tank filled with a photocurable liquid resin.
	Lubricant sublayer photo curing	A build plate inside a tank filled with liquid resin is exposed with UV light from underneath. Each exposure adds a whole layer to the build plate. The buid plate moves upwards after every light exposure. A lubricant is added to prevent the printed part from sticking to the membrane between the part and projector.
	Liquid-crystal display printing (LCD)	A build plate inside a tank filled with liquid resin is exposed with UV light from underneath. Areas which should not polymerize are covered from the curing light by a LCD screen.
	Continuous liquid interface production (CLIP)	A build plate inside a tank filled with liquid resin is exposed constantly with UV light from underneath and constantly moving upwards. An oxygen permeable membrane prevents the printed parts from sticking to the bottom of the tank.
extrusion of tough masses through nozzle	Fused deposition modeling (FDM) / fused filament fabrication (FFF)	A filament of a thermoplastic polymer is extruded through a hot nozzle.
melting/sintering/ binding of powder	Selective laser sintering (SLS)	A powder is spread layer by layer and sintered by a laser.
	Selective laser melting (SLM)	A metal powder is spread layer by layer and melted by a laser.
	Color jet printing (CJP)	Powder is spread layer by layer and (colored) binder is added through the printhead.

For all publications that evaluate printing errors, a subgroup analysis is performed for the basic printing technology used within every type of original structure

### Quantitative analysis

In a further step the literature is screened for measurements of linear deviations in medical 3D-printing processes. If average values are stated by the authors, the *absolute values of maximum mean deviation* (*AMMD*) per publication are reported. According to Eq. 1, the metric *AMMD* is defined as the absolute value of the largest avarage linear deviation. In Eq. 1, $\overline{x}$ represents avarage values of liner deviations and *i* = 1 to *i* = *n* the the number of parameters analyzed per publication.

1 $$AMMD={\mathit{\max}}_{i=1}^n\left(|{\overline{x}}_i|\right)$$

All values are reported individually for the main types of error (SegE, SegC, DEE, PrE, IAE) and their combinations, respectively. For the most frequently evaluated *partial error* (*printing error*, PrE) and the most frequently evaluated *combined error* (*total error,* SegE+DEE + PrE), a more detailed analysis is performed: AMMD are reported individually for each of their described sub-categories.

## Results

### Descriptive analysis

Since 2015, with seven publications meeting the inclusion criteria in that year, the amount of relevant publications has increased significantly. A preliminary peak is to be found in 2021, when 28 publications investigating geometric accuracy of medical 3D-printing were published. By December 4th 2022, a total of 139 investigations are identified. They are often specific to anatomical regions or artificial test specimen and are listed here according to descending prevalence: teeth or jaws without other bones of skull (*n* = 52), skull bones without jaw (*n* = 17), heart and coronary arteries (*n* = 16), artificial test specimen (*n* = 13). The following with less than *n* = 9 per region: bones of upper limb, brain vessels, bones of lower limb, pelvic bones, liver with bile ducts and / or gall bladder, thoracic aorta, kidney, animals, vertebrae or spine column, abdominal vessels, brain parenchyma, prostate, trachea or bronchial system, uterus, outer ear and nasal airways. By far the largest number of investigations are carried out in the field of dentistry (approximately one third of all included publications investigate structures that belong to the group “teeth or jaw without other skull bones“).

**Fig. 6:**
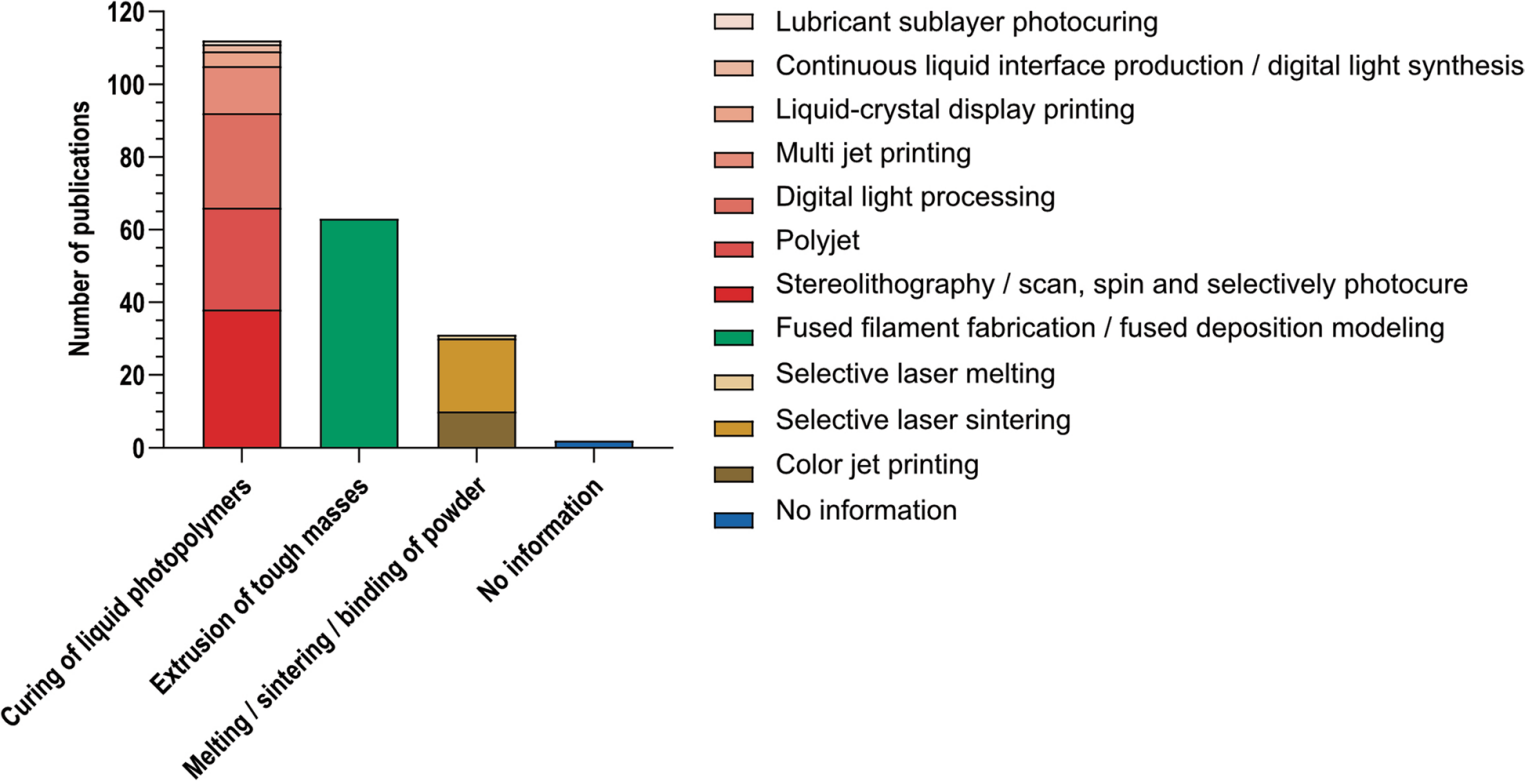
Printing technologies used within publications that assess the printing error. Absolute number of publications by printing technology as named by manufacturer of 3D-printer. Note that codominance of two or more printing technologies within one publication is possible

Publications evaluating printing errors are analyzed for the printing technologies used. Figure 6 shows the number of publications per proprietary printing technology for all of them as named by manufacturers. Figure 12 in Appendix B additionally shows the proportion of basic printing technologies per type of original structure.

Each publication is analyzed for the type of error evaluated, the measurement methods and the experimental setup of the image acquisition. Based on this analysis all publications are assigned to at least one of the defined categories. In the following section, the subcategories for the detailed analysis of the applied methods, together with the publications assigned to them, are shown for each main type of error and their possible combinations in Table 4 to Publications that evaluate the DEE and the PrE in combination are organized by applied methods and shown in Table 9**.** The same categories as described for the individual evaluation of the PrE are adopted. The only difference is that the direct result of segmentation is used as a reference instead of the print-STL.

**Table 4:** SegE evaluation. Publications according to the subcategories for the detailed analysis of applied methods as introduced in chapter 2.2.10. and visualized in Fig. 5. n.a. = not applicable, for subcategories no publication could be assigned to

Basic Approach	Imaging Setup	Tools for Linear Measurements	Time of Dissection	Publications
**lin**	**nosim**	cal		n.a.
		3Dcal		n.a.
	sim	cal		n.a.
		3Dcal		n.a.
	cad	cal	dis	[69]
			nodis	[15, 70, 71]
		3Dcal	dis	n.a.
			nodis	n.a.
	pat			n.a.
**surf**	nosim			n.a.
	sim			n.a.
	cad		dis	[72, 73]
			nodis	[74, 75]
**other**				[29, 74–77]

basic approach: linear (lin) or surface (surf) deviation based analysis; imaging setup: experimental setups of the image acqusition: nosim: artifical model and no simulation of adjacent tissue, sim: artifical model and simulation of adjacent tissue, cad: cadaver study, pat: scan of patients due to clinical questions; tools for linear measurements: cal: caliper, 3Dcal: 3D-scan and virtual linear measurements, pat: linear measurements on patient DICOM data; time of dissection (for cadaver studies): dissection is done before image acquisition (dis) or after image acquisition (nodis); could not be assigned to any of the introduced categories (other)

Table 9. Publications that evaluate the total error (SegE+DEE + PrE) are shown separately in Table 10.

Publications that evaluate the SegE individually are organized by applied methods and shown in Table 4.

Publications that evaluate the DEE individually are organized by applied methods and shown in Table 5.

**Table 5:** DEE evaluation. Publications according to the subcategories for the detailed analysis of applied methods as introduced in chapter 2.2.10. and visualized in Fig. 5

Basic Approach	Publications
**lin**	[70]
**surf**	[78]
**other**	[79]

basic approach: linear (lin) or surface (surf) deviation based analysis; could not be assigned to any of the introduced categories (other)

Publications that evaluate the PrE individually are organized by applied methods and shown in Table 6.

**Table 6:** PrE evaluation. Publications according to the subcategories for the detailed analysis of applied methods as introduced in chapter 2.2.10. and visualized in Fig. 5

Basic Approach	Tools for Linear Measurements	Publications
**lin**	**DICOM**	[80, 81]
	cal	[21, 69, 71, 82–101]
	3Dcal	[17, 31, 102–110]
**surf**		[17–19, 31, 85, 103, 105–128]
**other**		[129–141]

basic approach: linear (lin) or surface (surf) deviation based analysis; tools for linear measurements: DICOM: CT-Scan of 3D-printed model and linear measurements on resulting DICOM data, cal: caliper, 3Dcal: 3D-scan and virtual linear measurements; could not be assigned to any of the introduced categories (other)

Publications that evaluate the IAE and SegC individually are shown in Table 7**.**

**Table 7:** IAE and SegC assessment

Main Types of Error	Publications
IAE	[15, 70, 71, 91, 100, 119, 126, 127, 142–146]
**SegC**	[126, 147–150]

Publications that evaluate the SegE and the DEE in combination are organized by applied methods and shown in Table 8. The same categories as described for the individual evaluation of the SegE are adopted. The only difference is that the print-STL is compared with the original structure instead of the direct segmentation result.

**Table 8:** combined evaluation of SegE and DEE. Publications according to the subcategories for the detailed analysis of applied methods as introduced in chapter 2.2.10. and visualized in Fig. 5. n.a. = not applicable, for subcategories no publication could be assigned to

Basic Approach	Imaging Setup	Tools for Linear Measurements	Time of Dissection	Publications
**lin**	**nosim**	cal		[151, 152]
		3Dcal		n.a.
	sim	cal		n.a.
		3Dcal		n.a.
	cad	cal	dis	[153]
			nodis	n.a.
		3Dcal	dis	n.a.
			nodis	n.a.
	pat	pat		[80, 81, 91, 95, 129, 134, 154, 155]
**surf**	nosim			n.a.
	sim			n.a.
	cad		dis	[156]
			nodis	n.a.
**other**				n.a.

basic approach: linear (lin) or surface (surf) deviation based analysis; imaging setup: experimental setups of the image acqusition: nosim: artifical model and no simulation of adjacent tissue, sim: artifical model and simulation of adjacent tissue, cad: cadaver study, pat: scan of patients due to clinical questions; tools for linear measurements: cal: caliper, 3Dcal: 3D-scan and virtual linear measurements, pat: linear measurements on patient DICOM data; time of dissection (for cadaver studies): dissection is done before image acquisition (dis) or after image acquisition (nodis); could not be assigned to any of the introduced categories (other)

Publications that evaluate the DEE and the PrE in combination are organized by applied methods and shown in Table 9. The same categories as described for the individual evaluation of the PrE are adopted. The only difference is that the direct result of segmentation is used as a reference instead of the print-STL.

**Table 9:** Combined evaluation of DEE and PrE. Publications according to the subcategories for the detailed analysis of applied methods as introduced in chapter 2.2.10. and visualized in Fig. 5. n.a. = not applicable, for subcategories no publication could be assigned to

Basic Approach	Tools for Linear Measurements	Publications
**lin**	**DICOM**	n.a.
	cal	n.a.
	3Dcal	n.a.
**surf**		n.a.
**other**		[157]

basic approach: linear (lin) or surface (surf) deviation based analysis; tools for linear measurements: DICOM: CT-Scan of 3D-printed model and linear measurements on resulting DICOM data, cal: caliper, 3Dcal: 3D-scan and virtual linear measurements; could not be assigned to any of the introduced categories (other)

According to our categorization, SegE experiments are categorized based on two key factors: 1. the measurement method employed, such as surface deviation analysis or linear measurements and 2. the imaging setup, which involves, among other aspects, distinguishing between phantom or cadaver studies.

In contrast, when subdividing experiments to assess the PrE, only the measurement methods used are considered, as the imaging setup does not influence this aspect. Conversely, subdividing experiments that evaluate the *total error* is more intricate due to the involvement of three key factors: 1. The measurement tools employed to assess the original structure; 2. the imaging setup used and 3. the measurement tools employed to evaluate the printed model.

**Fig. 7:**
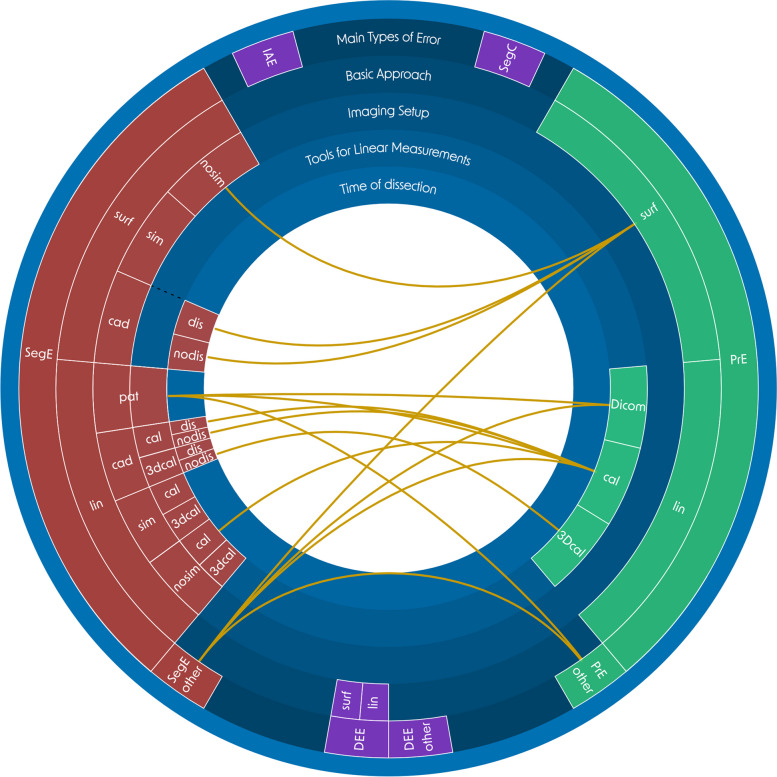
Total error assessment. All combinations of SegE subcategories (describing methods for measuring the original structures and setups of the image acquisition) with PrE subcategories (describing methods for measuring the printed models), which are found in the included literature. Basic approach: linear (lin) or surface (surf) deviation based analysis; imaging setup: experimental setups of the image acqusition: nosim: artifical model and no simulation of adjacent tissue, sim: artifical model and simulation of adjacent tissue, cad: cadaver study, pat: scan of patients due to clinical questions; tools for linear measurements: DICOM: CT-Scan of 3D-printed model and linear measurements on resulting DICOM data, cal: caliper, 3Dcal: 3D-scan and virtual linear measurements, pat: linear measurements on patient DICOM data; time of dissection (for cadaver studies): dissection is done before image acquisition (dis) or after image acquisition (nodis); could not be assigned to any of the introduced categories (other)

Given that the SegE sub-categories encompass the first two key factors and the PrE subcategories encompass the third one, they can be integrated to form total error subcategories that encompass all three key factors. The complexity arising from the various combinations of SegE and PrE subcategories found in the literature is illustrated in (Fig. 7).

Table 10 shows all combinations of SegE subcategories (describing methods for measuring the original structures and setups of image acquisition) with PrE subcategories (those describe methods for measuring the printed model) found in the literature (Fig. 7). For each combination, the corresponding publications are listed.

**Table 10:** total error (SegE+DEE + PrE) evaluation. Publications according to the combinations of subcategories of the segmentation error (describing methods for measuring the original structures and setups of image acquisition) and the printing error (describing methods for measuring the printed model). All combinations found in the literature are listed. Combinations no publications could be assigned to are not shown

Segmentation Error				Printing Error		Publications
basic approach	imaging setup	tools for linear measurements	time of dissection	basic approach	tools for linear measurements	
**lin**	**nosim**	**cal**		lin	cal	[121, 134, 144, 152, 158–160]
	cad	cal	dis			[153, 161–166]
		cal	nodis			[15, 70, 71, 167]
		3Dcal	nodis		3Dcal	[168]
	pat				DICOM	[80, 81, 137, 141, 169–178]
	pat				cal	[95, 146, 154, 155, 179–190]
	pat			other		[129, 142, 171, 191–194]
surf	nosim			surf		[119, 195–198]
	cad		dis			[72]
			nodis			[125]
other				lin	DICOM	[199]
					cal	[93, 99, 100, 145, 200]
				surf		[201]
				other		[142]

basic approach: linear (lin) or surface (surf) deviation based analysis; imaging setup: experimental setups of the image acqusition: nosim: artifical model and no simulation of adjacent tissue, sim: artifical model and simulation of adjacent tissue, cad: cadaver study, pat: scan of patients due to clinical questions; tools for linear measurements: DICOM: CT-Scan of 3D-printed model and linear measurements on resulting DICOM data, cal: caliper, 3Dcal: 3D-scan and virtual linear measurements, pat: linear measurements on patient DICOM data; time of dissection (for cadaver studies): dissection is done before image acquisition (dis) or after image acquisition (nodis); could not be assigned to any of the introduced categories (other)

### Quantitative analysis

**Fig. 8:**
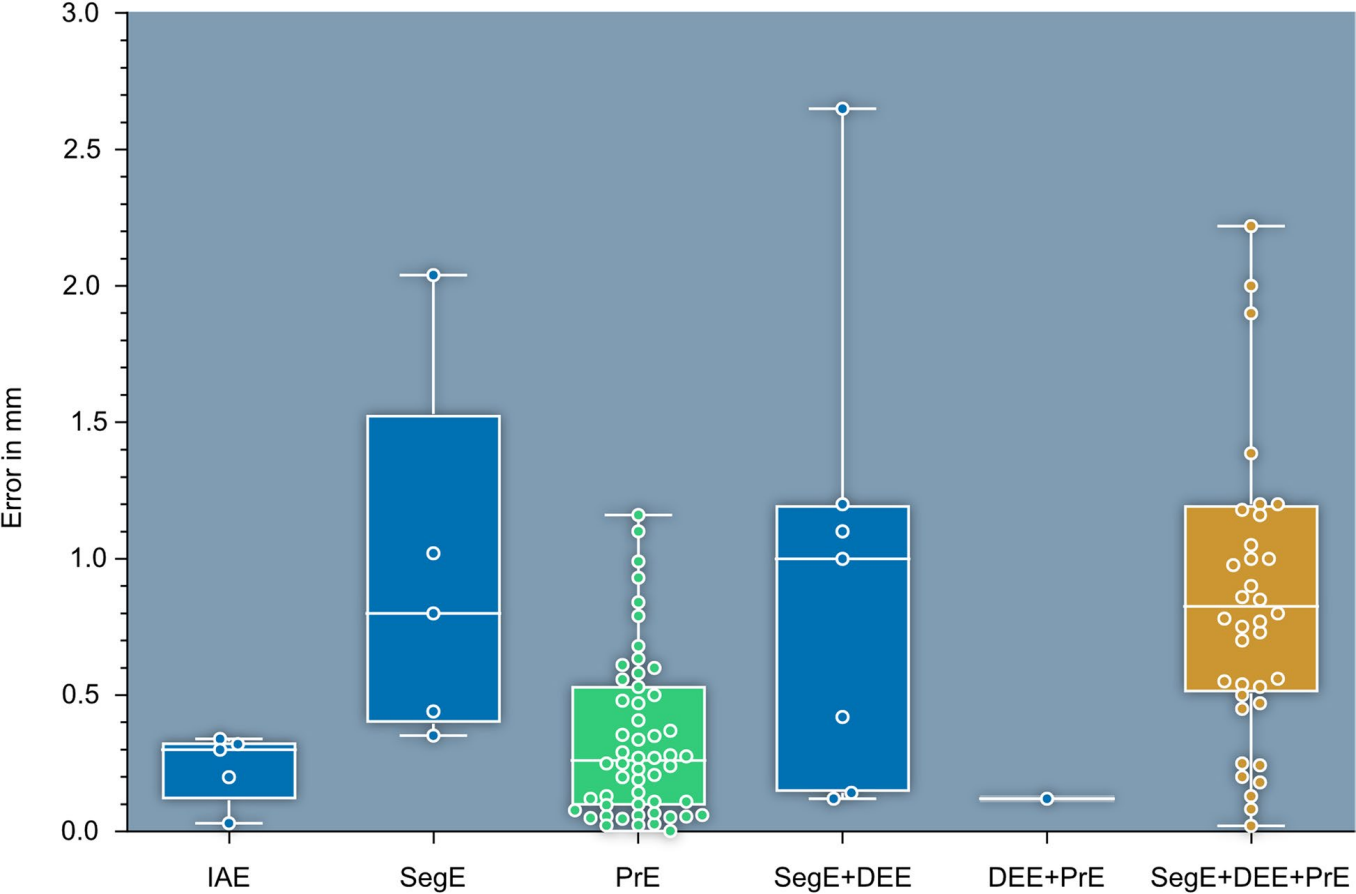
AMMD, organized by main types of error and their combinations. IAE: *Image Acquistion Error*, SegE: *Segmentation Error*, DEE: *Digital Editing Error*, PrE: *Printing Error*. PrE (green) is the most frequently evaluated *partial error* and SegE+DEE + PrE (yellow) is the most frequently evaluated *combined error*

The *absolute values of maximum mean deviations* per publication (AMMD) are illustrated for each main type of error in Fig. 8. No mean values are found for the digital editing error. Mean values are not computed - only the values explicitly reported by the authors are presented. All five outliers are not displayed in Fig. 8: one printing error outlier (PrE) with a value of 6.44 mm [141] and four total error outliers (SegE+DEE + PrE) with values of 3.0 mm [165], 3.8 mm [183], 3.81 mm [95] and 4.8 mm [170].

**Fig. 9:**
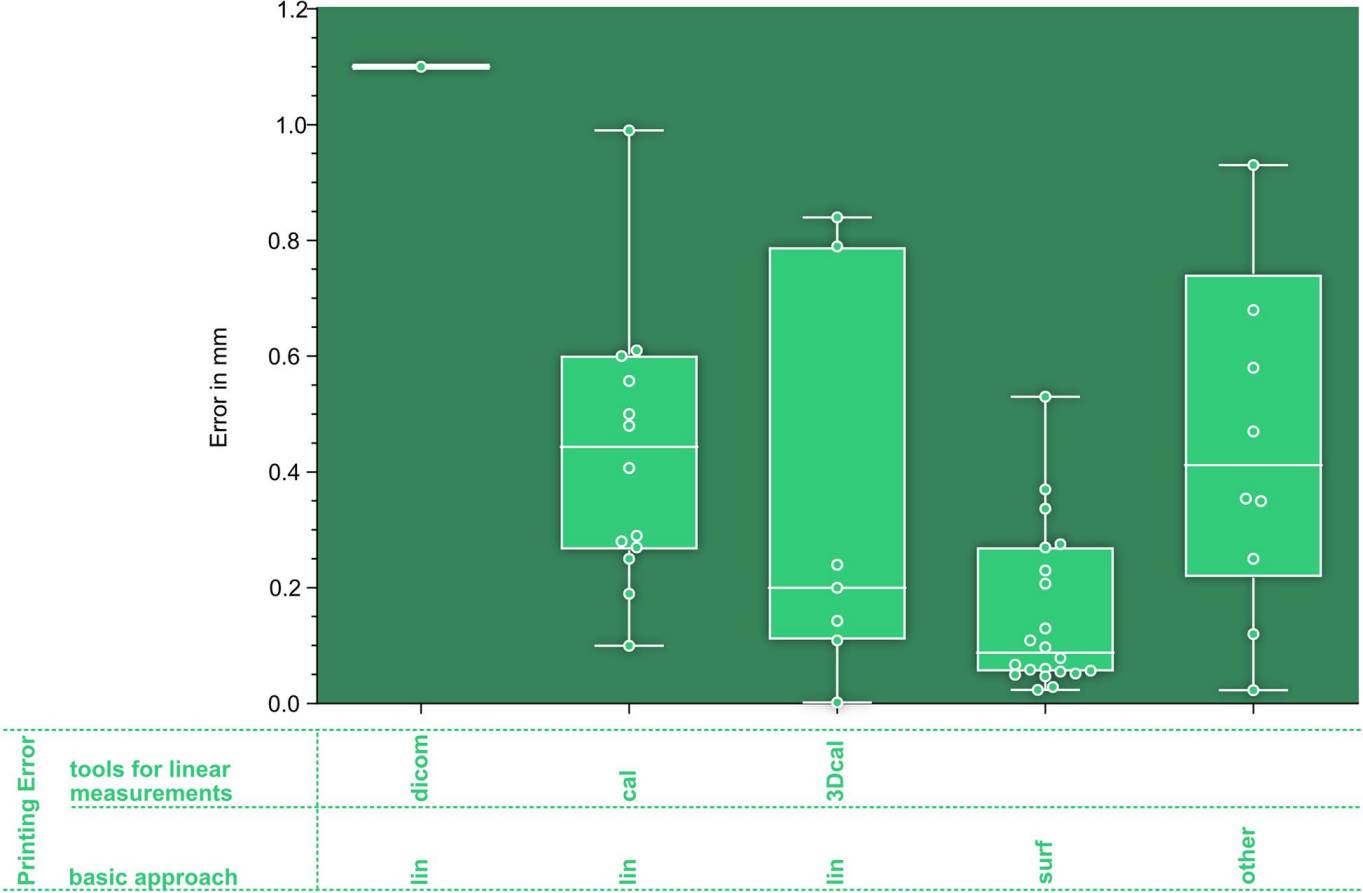
Evalutation of printing errors. AMMD, organized by subcategories of the PrE. basic approach: linear (lin) or surface (surf) deviation based analysis; tools for linear measurements: DICOM: CT-Scan of 3D-printed model and linear measurements on resulting DICOM data, cal: caliper, 3Dcal: 3D-scan and virtual linear measurements; could not be assigned to any of the introduced categories (other)

The green box in Fig. 8 shows the AMMD for the printing error. A more detailed analysis of the printing error including the subcategories can be found in Fig. 9. All three outliers are not displayed in Fig. 9: One caliper group outlier (PrE_lin_cal) with a value of 1.16 mm [95], one 3D-scan and surface comparison group outlier (PrE_surf) with a value of 0.633 mm [113] and one outlier in the group of heterogenous approaches (PrE_other) with a value of 6.44 mm [141].

**Fig. 10:**
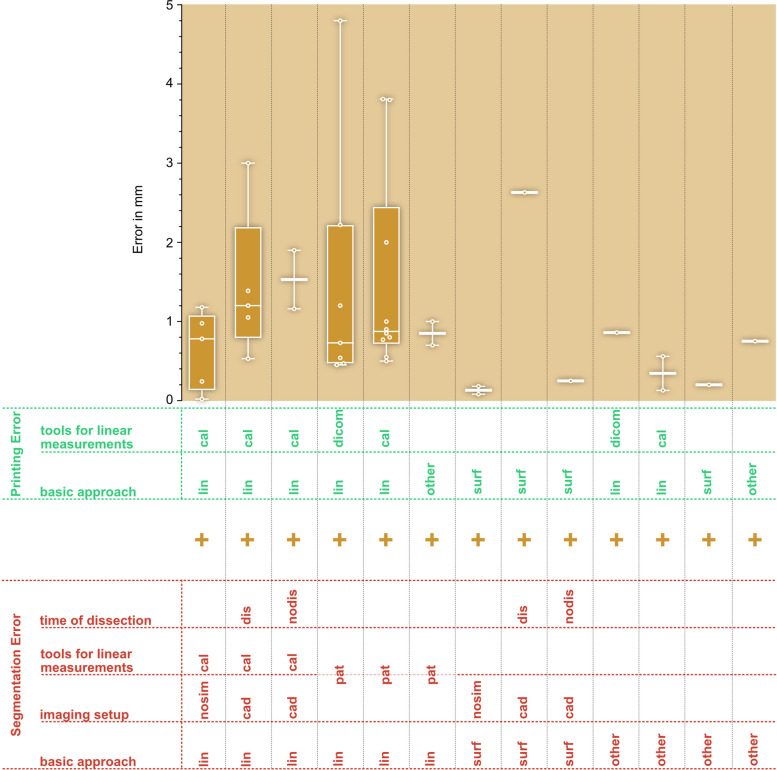
Evalutation of total errors. AMMD, organized by combination of subcategories of the *segmentation error* (describing methods for measuring the original structures and setups of image acquisition) and the *printing error* (describing methods for measuring the printed model) as shown in Fig. 7 and Table 10 basic approach: linear (lin) or surface (surf) deviation based analysis; imaging setup: experimental setups of the image acqusition: nosim: artifical model and no simulation of adjacent tissue, sim: artifical model and simulation of adjacent tissue, cad: cadaver study, pat: scan of patients due to clinical questions; tools for linear measurements: DICOM: CT-Scan of 3D-printed model and linear measurements on resulting DICOM data, cal: caliper, 3Dcal: 3D-scan and virtual linear measurements, pat: linear measurements on patient DICOM data; time of dissection (for cadaver studies): dissection is done before image acquisition (dis) or after image acquisition (nodis); could not be assigned to any of the introduced categories (other)

The yellow box of Fig. 8 shows the AMMD for the *total error* (SegE+DEE + PrE). A more detailed analysis of the total error including the combinations of SegE and PrE subcategories can be found in Fig. 10.

## Discussion

In terms of quality assurance in medical 3D-printing the field of dentistry accounts for the largest proportion of all publications (37%). This is primarily due to the relatively long history of CAD/CAM (Computer-Aided Design/Computer-Aided Manufacturing) processes in dentistry. A historical perspective of CAD/CAM development in dentistry is provided by Miazaki et al., starting in 1971 with the utilization of computer-guided subtractive manufacturing machines for crown production based on optical impressions [202]. The CAD/CAM process then paved the way for the integration of additive manufacturing techniques in dentistry, earlier than in other medical fields. Rekow has also highlighted the role of additive manufacturing in “digital dentistry” [203].

In our findings liquid photopolymer curing is generally the most employed basic printing technology. In dentistry, the use of SLA (Stereolithography), DLP (Digital Light Processing), or PJP (PolyJet Printing) is prevalent. These methods offer advantages such as short production times, high surface resolution, and mechanical durability [204]. FFF (Fused Filament Fabrication) represents the largest category of individual technologies. Overall, FFF-based processes are the most cost-effective and easiest to use. They are well-suited for initial exploration of 3D-printing and at the same time enable the production of finalized products and sterilizable implants [205]. In contrast, the least studied techniques are powder-based. This is likely because they are cost-intensive and most complex to implement. Their application requires a substantial allocation of resources and specific technical expertise, which can be limited in clinical settings. Illi et al. provided a comprehensive overview of a cardiovascular phantom production process, including reporting on the 10 largest studies concerning geometric accuracy [14]. Among these, material jetting and stereolithography are the most widely used 3D-printing technologies, which is consistent with our findings.

Among the *partial errors*, DEE as *digital editing error* is the least studied, while PrE as *printing error* is the most studied. Nevertheless, our results allow for an initial assessment of DEE in terms of its impact on the *total error*. The SegE was even studied significantly less often than the PrE, although the SegE seems to be much more influenced by manual input. When assessing the segmentation process, a distinction is made between publications in which the direct segmentation result was compared to the original structure (SegE) and those in which the segmentation was further processed (SegE+DEE), for example, using digital editing techniques such as smoothing. Analyzing the deviations in both groups, their values do not differ greatly. This may indicate that standard digital editing techniques such as smoothing or artefact removal could have a minor impact on the *total error* of the production process. This assumption is supported by results from Ionata et al. presenting the only available AMMD for the combination of *digital editing error* and *printing error* (DEE + PrE): with a value of 0.12 mm [157], it is close to the lower end of the range of values obtained for the isolated printing error (PrE). However, further research is needed to quantify the influence of DEE, as only three publications are found that individually address this error.

*Segmentation Error* (SegE) is often evaluated using linear measurements. However, in the literature it is often not clear whether the original structure was compared to the direct segmentation result or to the *print-STL*. For future investigations, it would be beneficial to strive for more concise reporting. Our categorization of SegE is based on two dimensions: the measurement methods used and the imaging setup, enabling an assessment of the applied methods in terms of accuracy and realism:

The least accurate measurement method is the comparison of linear measurements on multi-planar reconstructions of slice images with corresponding linear measurements of the segmentation result (SegE_lin_pat). The most accurate measurement method is surface deviation analysis between a 3D-scan of the original structure and the segmentation result.

**Fig. 11:**
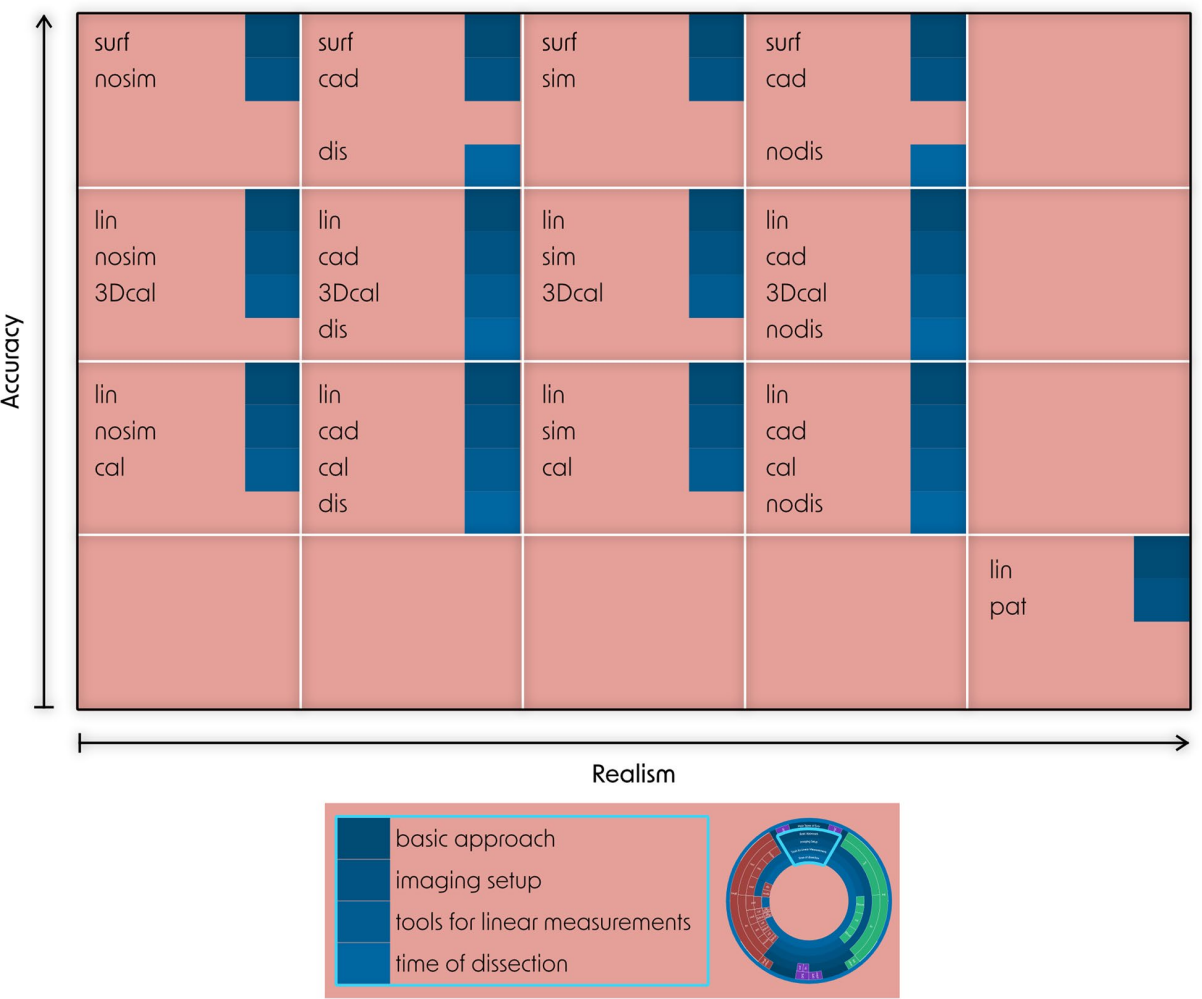
Accuracy and realism of SegE subcategories, according to the subcategories for the detailed analysis of applied methods as introduced in chapter 2.2.10. and visualized in Fig. 5. Accuracy is mainly influenced by the basic approach and tools for linear measurements, while realism is mainly influenced by imaging setup and time of dissection. Basic approach: linear (lin) or surface (surf) deviation based analysis; imaging setup: experimental setups of the image acqusition: nosim: artifical model and no simulation of adjacent tissue, sim: artifical model and simulation of adjacent tissue, cad: cadaver study, pat: scan of patients due to clinical questions; tools for linear measurements: cal: caliper, 3Dcal: 3D-scan and virtual linear measurements, pat: linear measurements on patient DICOM data; time of dissection (for cadaver studies): dissection is done before image acquisition (dis) or after image acquisition (nodis)

**Fig. 12:**
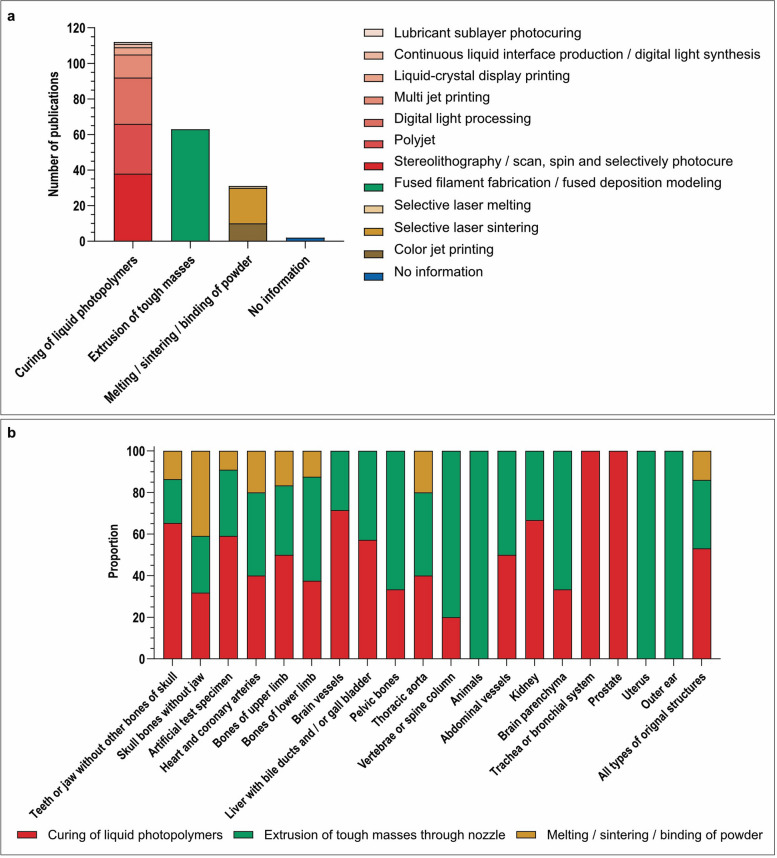
Used printing technologies within publications that assess the printing error. **a** absolute number of publications by printing technology as named by manufacturer of 3D-printer. **B** proportion of basic 3D-printing technologies by types of original structures. Right column: overall proportion of basic 3D-printing technologies. The groups “curing of liquid photopolymers”, extrusion of tough masses through nozzle” and “melting/sintering/binding of powder“are formed according to the main printing technologies as described in Table 3. Note that codominance of two or more printing technologies within one publication is possible

The least realistic imaging setup is scanning artificial models in air, while the most realistic one involves scanning real patients for a clinical assessment (Fig. 11).

George et al. noted that the deviation of the segmentation process may be significantly lower when the original structures are scanned in air compared to in situ image acquisition [67]. This can pose a challenge: The most realistic imaging setup can only be combined with the least accurate measurement method. Therefore, a viable compromise is desirable. Cadaver studies have frequently been employed for this purpose as gold-standard [15, 69, 74]. These involve the dissection of soft tissue before or after imaging to allow reference measurements on the target structure of the segmentation (*original structure*). However, cadaver studies are expensive, personnel-intensive and need ethical approval in advance.

An accurate and realistic assessment of SegE can generally be defined as follows: A complex three-dimensional (anatomical) structure is surrounded by randomly arranged isodense structures during imaging. It is then segmented, and a 3D-scan of the original structure is compared with the direct segmentation result via surface comparison. Based on this definition, cadaver studies may not be the optimal method to evaluate SegE, considering the enormous efforts. To overcome these obstacles, future endeavours could focus on developing simple segmentation models that use artificial (3D-printed anatomical) structures and simulate adjacent (soft-) tissue.

Regarding combined errors, the combination of DEE and PrE has been studied the least, while the *total error* has been studied the most. Since the *total error* includes SegE, among others, similar challenges arise. Figure 7 illustrates methods for evaluating the *total error* that are found in the literature. They range from simple and inexpensive methods (e.g. scanning an artificial model in air combined with a comparison of linear measurements taken on the original structure and the printed model) to the current gold-standard (scanning of cadveric specimen, combined with a surface comparison between a 3D-scan of the *original structure* after dissection and a 3D-scan of the *printed model*). Besides cost, personnel and ethical aspects a limited availability has to be considered, too.

As a consequence, the need for alternatives seems justified. The combination of an artificial original structure with simulated adjacent tissue could be a promising solution that can be expected to achieve comparable accuracy and realism to cadaver studies but with significantly reduced cost and effort. Some attempts have been described in the literature to simulate adjacent tissue to increase the realism of the segmentation process [77, 156]. However, those still involve the use of cadaveric specimens: Van Eijnatten et al. embedded a human dry skull in silicone to simulate soft tissue for validating the influence of the head position during cone-beam CT [156]. Zhang et al. [77] placed artificial models of nasal airways inside a dry skull to use them as a realistic benchmark for the validation of their segmentation algorithm.

Judging by literature, the impression is that the preferred methods to investigate the *total error* are based on linear measurements on DICOM datasets of real patients. These methods offer the advantage that no experimental imaging is required. Instead, one can easily access the clinical database after obtaining ethical approval to use existing data. Nevertheless, methods involving patient data are associated with a serious weakness: reference measurements are limited to the least accurate method (linear measurements on multi planar reconstructions).

The data points in Fig. 8 are referenced to different publications. However, their deviation values for the *total error* are not significantly larger than for the *partial errors*. This may indicate that some of the partial errors compensate for each other. This is particularly relevant in a clinical environment where numerous specialist disciplines are often involved in the production of a 3D-printed patient-specific anatomical model, especially when considering that in some cases not all process steps may be the responsibility of the technician. For instance, technicians could have two options for digital editing: 1. They receive the DICOM dataset and perform the segmentation themselves, or 2. they receive a completed segmentation from a clinician. Assuming the latter, a technician could document a very high result quality for the production process, e.g. because it relies on a significantly positive SegE and a significantly negative PrE. The expected high quality of results would no longer be guaranteed if the segmentation generates a strongly negative SegE. It can be concluded that assessing process quality solely based on the total error without knowledge of the partial errors is insufficient.

Although only one AMMD was found for the PrE_lin_DICOM group in Fig. 8, it provides an indication of the hierarchy of measurement accuracies for PrE determination. The dispersion and the median values of the measurements decrease from left to right, corresponding to an increase in the accuracy of the measuring instrument. Linear measurements on DICOM datasets are the least accurate, surface deviation analyses are the most accurate. Nevertheless, it is important to consider that reporting the mean deviation of a surface deviation analysis alone tends to overestimate precision, as the value might be low even if the evaluated *process precision* itself is poor. This can happen when a high number of measurement points can lead to the summative elimination of positive and negative deviations. The discriminatory power of a surface deviation analysis can be significantly improved when the surface proportion within a tolerance based on clinical requirements is reported, as demonstrated by Lo Giudice et al. [148], Jin et al. [198] and Akyalcin et al. [103]. Specific tolerances for 3D-printing, tailored to anatomical regions and clinical demands, could be derived from minimal requirements for the registration in computer-assisted surgery (“navigation”), which range from 0.5 mm for spinal screw insertion to 2 mm in pelvic bone tumor resection [206–208].

That aspect is further illustrated in Fig. 10: Although only a small number of values is found for the group “SegE_surf_nosim - PrE_surf” their median value and dispersion are low. This group involves image acquisition of artificial models in air and a surface comparison between the 3D-scan of the *original structure* and the 3D-scan of the *printed model*. As a result, the least realistic imaging setup, with its associated minimal segmentation error, is combined with the measurement tool that tends to produce the lowest values.

Generally, the deviations are within the low single-digit millimeter range, which agrees with findings from Chae et al. They focused on 19 publications that evaluate the accuracy of medical 3D-printing using cadaveric specimens and linear measurements [15].

On the level of main types of errors five outliers are not displayed in Fig. 8. One PrE outlier (green box) is not displayed which is presented by Witowski et al., with a value of 6.44 mm [141]. Two reasons may explain this higher value compared to the rest of the literature: Firstly, their production process appears to be prone to potential inaccuracies (3D-printing of multi-part moulds, followed by assembly of the moulds and casting with silicone [209]). Secondly, they employ a measurement method of questionable accuracy: They acquire CT scans of the models, segment them and then, after alignment, perform a surface comparison between the patient segmentation and the model segmentation. Figure 8 shows that the variance and the median values are larger for segmentation errors (SegE) than for printing errors (PrE). The error associated with the measurement principle (image acquisition followed by segmentation and surface comparison) appears to be larger than the expected error size here. A similar approach was chosen by other authors [130, 132, 134, 135, 137]. However, it is generally questionable whether slice image acquisition followed by segmentation is a valid tool to perform a surface deviation-based evaluation of the printing error. The same applies to the comparison of linear measurements between patient and model segmentation as described by Liang et al. [135].

Four total error outliers (Fig. 8, yellow box) are not displayed: those are presented by Silva et al. [165], Larguier et al. [183], Perica et al. [95] and Hedelin et al. [170] with values of 3.0 mm, 3.8 mm, 3.81 mm and 4.8 mm, respectively.

Several reasons might contribute to those comparatively high deviations: Some landmarks are difficult to identify for caliper measurements. In particular, if the model is scaled down for the 3D-print, this may result in a limited accuracy of the measurements. Larguier et al. for example validated their measurements in terms of accuracy and stated: “The caliper measurements of CD showed only moderate accuracy” [183]. Another aspect may be that the variety of dental publications leads to a relatively small average of measurements. As the focus is on absolute errors, which tend to be larger for bigger measurements, this could be an explanation for the relatively high value of some outliers. Future research may extend the present review to the analysis of relative errors.

Odeh et al. [210] defined checkpoints within the medical 3D-printing process at which measurements should be taken for quality assurance. They evaluated the *combined error* of segmentation and digital editing (SegE+DEE) as well as the *printing error* (PrE) and the *total error* (SegE+DEE + PrE). The same “checkpoints” are applied by Allan et al. and Perica et al. [80, 95]. Nevertheless, it should be noted that both only printed one model, which may limit the significance of their results.

In summary, there are various challenges in quality assurance for patient-specific 3D-printed anatomical models. These challenges can lead to either an overestimation or underestimation of the investigated errors. Particularly large or small values do not necessarily indicate exceptionally high or low accuracy; instead, the influence of the methods used for the evalutation should be critically examined. Future research should focus on developing realistic and resource-efficient segmentation models that also allow for high-accuracy measurements.

Recently published and upcoming standards address the early stage of maturity of nearly all additive manufacturing process categories, still resulting in strong deviations in some cases and a significant human-based error factor potential. The typical processual error reasons are missing process quality assurance, deviations in hardware, software, environmental conditions or feedstock. Therefore, the validation for accuracy should be part of the validation procedure. To assist this the first ISO/ASTM standards are published, e.g., ISO/ASTM 52901, 52,920, 52,930, 52,907, 529,004. Together with this review those will build a fundamental basis for a standardized qualification of the entire workflow.

### Limitations

The literature search and the literature screening process was conducted by a single individual. The influence of the year of publication, the 3D-printing technology used as well as the image acquisition modalities or parameters on the geometric accuracy are not evaluated, which may be a relevant topic for future research. The publication focused solely on printing technologies and did not address specific printer models and their manufacturers. An interesting future research project could aim to investigate whether printers with 510(k) clearance are more accurate than printers without. The predominance of processes or printing technologies with a particularly high or low accuracy within some of our subcategories may cause bias for the deviation values.

## Conclusions

This systematic review is an attempt to classify the literature regarding quality assurance of the geometric accuracy of patient-specific 3D-printed anatomical models into comparable categories. These are based on the measurement methods used and the experimental setups of the image acquisition.

In general, experimentally determined total errors do not appear to be significantly larger than partial errors. This suggests that partial errors may cancel each other out. Future research should therefore aim to investigate partial errors experimentally to describe the total error as the sum of the partial errors according to the rules of error propagation.

Current methods for quality assurance of the segmentation are either realistic and accurate or resource efficient. Future research should focus on implementing models that allow for evaluations with high accuracy and realism while being easy and cheap to perform. Those could also be used for further evaluation of influences of imaging parameters on the segmentation error.

Our system of categorization may be a valuable contribution to the structural design and reporting of future experiments as well as enhance the understanding of the overall process, not only for clinicians. It could support the training of specialists for risk assessment and process validation within the additive manufacturing industry.

### Supplementary Information

**Additional file 1:.** High resolution version of the graphical abstract.

## Data Availability

No datasets were generated or analysed during the current study.

## Appendix 1

### Evaluation of the Segmentation Error (SegE)

In the following methods are described which evaluate the SegE on basis of linear landmark measurements **(SegE_lin)**:

Medical images of an artificial structure (e.g. a 3D-printed anatomical model) are acquired without the simulation of the adjacent (soft) tissue. The structure is then segmented on the resulting DICOM images, and the original structure is compared with the segmentation result **(SegE_lin_nosim)**.

Linear measurements of landmarks are taken directly on the original structure with a caliper or a similar measuring tool and are compared with the corresponding digital measurements of the segmentation result **(SegE_lin_nosim_cal)**.

The original structure is 3D-scanned with an optical scanning system and digital linear measurements of landmarks are compared with the corresponding digital measurements of the segmentation result **(SegE_lin_nosim_3Dcal)**.

Medical images of an artificial structure (e.g. a 3D-printed anatomical model) are acquired with the simulation of the adjacent (soft) tissue. The structure is then segmented on the resulting DICOM images, and the original structure is compared with the segmentation result **(SegE_lin_sim)**.

Linear measurements of landmarks are taken directly on the original structure with a caliper or a similar measuring tool and are compared with the corresponding digital measurements of the segmentation result **(SegE_lin_sim_cal)**.

The original structure is 3D-scanned with an optical scanning system and digital linear measurements of landmarks are compared with the corresponding digital measurements of the segmentation result **(SegE_lin_sim_3Dcal)**.

Medical images of a cadaveric specimen are acquired. The cadaveric specimen is then segmented on the resulting DICOM images, and the original cadaver is compared with the segmentation result. To measure landmarks of the cadaver, it must be dissected. This can be done either prior to the image acquisition or after the image acquisition **(SegE_lin_cad)**.

The dissection is done prior to the image acquisition. Linear measurements of landmarks are taken directly on the cadaver with a caliper or a similar measuring tool and are compared with the corresponding digital measurements of the segmentation result **(SegE_lin_cad_ cal_dis)**.

The dissection is done after the image acquisition. Linear measurements of landmarks are taken directly on the cadaver with a caliper or a similar measuring tool and are compared with the corresponding digital measurements of the segmentation result **(SegE_lin_cad_cal_nodis)**.

The dissection is done prior to the image acquisition. The structures of interest of the cadaver are 3D-scanned with an optical scanning system and digital linear measurements of landmarks are compared with the corresponding digital measurements of the segmentation result **(SegE_lin_cad_3Dcal _dis)**.

The dissection is done after the image acquisition. The structures of interest of the cadaver are 3D-scanned with an optical scanning system and digital linear measurements of landmarks are compared with the corresponding digital measurements of the segmentation result **(SegE_lin_cad_3Dcal _nodis)**.

Segmentation is conducted on original patient DICOM data and landmarks are measured on those. Linear measurements on DICOM data are compared with the corresponding digital measurements of the segmentation result **(SegE_lin_pat)**.

In the following methods are described which evaluate the SegE on basis of a surface deviation analysis. **(SegE_surf)**:

Medical images of an artificial structure (e.g. a 3D-printed anatomical model) are acquired without the simulation of the adjacent (soft) tissue. The structure is then segmented on the resulting DICOM images and the original structure is compared with the segmentation result. The original structure is 3D-scanned with an optical scanning system and the resulting digital model of the original structure is then compared with the segmentation result through surface deviation analysis after alignment **(SegE_surf_nosim)**.

Medical images of an artificial structure (e.g. a 3D-printed anatomical model) are acquired with the simulation of the adjacent (soft) tissue. The structure is then segmented on the resulting DICOM images and the original structure is compared with the segmentation result. The original structure is 3D-scanned with an optical scanning system and the resulting digital model of the original structure is then compared with the segmentation result through surface deviation analysis after alignment **(SegE_surf_sim)**.

Medical images of a cadaveric specimen are acquired. The cadaveric specimen is then segmented on the resulting DICOM images and the original cadaver is compared with the segmentation result. To perform a 3D scan of the cadavers’ structures of interest, it needs to be dissected. This can be done either prior to the image acquisition or after the image acquisition **(SegE_surf_cad)**.

The dissection is done prior to the image acquisition. The structures of interest of the cadaver are 3D-scanned with an optical scanning system. The resulting digital model of the cadaver is then compared with the segmentation result through surface deviation analysis after alignment **(SegE_surf_cad_dis)**.

The dissection is done after the image acquisition. The structures of interest of the cadaver are 3D-scanned with an optical scanning system. The resulting digital model of the cadaver is then compared with the segmentation result through surface deviation analysis after alignment **(SegE_surf_cad_nodis)**.

Although this categorization approach covers the majority of experiments, some cannot be assigned to one of the categories. Those are summarized within the category **SegE_other**.

### Evaluation of the Digital Editing Error (DEE)

Digital linear measurements of landmarks of the direct segmentation result are compared with corresponding digital linear measurements of the print-STL **(DEE_lin)**.

Segmentation result and print-STL are compared through surface deviation analysis after alignment **(DEE_surf)**.

Although this approach for categorization covers the majority of experiments some cannot be assigned to one of the categories. Those are summarized within the category **DEE_other**.

### Evaluation of the Printing Error (PrE)

In the following methods are described which evaluate the PrE on basis of linear landmark measurements **(PrE_lin)**:

CT scans of the printed model are acquired. Digital linear measurements of landmarks of the print-STL are compared with corresponding linear measurements on DICOM data of the printed model **(PrE_lin_DICOM)**.

Digital linear measurements of landmarks of the print-STL are compared with corresponding direct measurements of the printed model, taken using a caliper or a similar measuring tool **(PrE_lin_cal)**.

The printed model is 3D-scanned with an optical scanning system. Digital linear measurements of landmarks of the print-STL are compared with corresponding digital linear measurements of the printed model **(PrE_lin_3Dcal)**.

In the following methods are described which evaluate the PrE on basis of a surface deviation analysis:

The printed model is 3D-scanned with an optical scanning system. The resulting digital model of the printed model is then compared with the print-STL through surface deviation analysis after alignment **(PrE_surf)**.

Although this categorization approach covers the majority of experiments, some cannot be assigned to one of the categories. Those are summarized within the category **PrE_other**.

### Evaluation of the Image Acquisition Error (IAE)

Medical images of an original structure are acquired. Linear measurements of landmarks are taken directly on the original structure with a caliper or a similar measuring tool and are compared with the corresponding linear measurements on DICOM data **(IAE)**.

### Segmentation Comparison Error (SegC)

Comparison of linear measurements of landmarks of different segmentation results with each other or comparison with each other through surface deviation analysis after alignment **(SegC)**.

### Combination of Segmentation Error and Digital Editing Error (SegE+DEE)

The combined error of segmentation and digital editing can be determined by measuring the original structure and comparing it to the print-STL. To achieve a more detailed classification the same categories as described for the individual evaluation of the segmentation error are adopted. The only difference is that the print-STL is compared with the original structure instead of the direct segmentation result.

If the authors did not specify whether they compared the original structure with the direct segmentation result or with the print-STL, it is assumed that they evaluated the segmentation error individually.

### Combination of Digital Editing Error and Printing Error (DEE + PrE)

The combined error of digital editing and printing can be determined by measuring the direct segmentation result and comparing it to the printed model. To achieve a more detailed classification the same categories as described for the individual evaluation of the printing error are adopted. The only difference is that the direct result of segmentation is used as a reference instead of the print-STL.

### Combination of Segmentation Error, Digital Editing Error and Printing Error (SegE+DEE + PrE)

The combined error of segmentation, digital editing and printing can be determined by measuring the original structure and comparing it with the printed model. These methods determine the overall error of the entire process. To achieve a more detailed classification, it is necessary consider methods for measuring both the original structure and the printed model. Additionally, it is important to consider the experimental setup of the image acquisition. Categories as described for the individual evaluation of the segmentation error describe methods to measure the original structure and the experimental setup of the image acquisition. Categories as described for the individual evaluation of the printing error describe methods to measure the printed model. Therefore, subcategories of segmentation errors and printing errors can be combined to provide a more detailed classification of measurements of the total error.

## Abbreviations

SegE: Segmentation error
DEE: Digital editing error
PrE: Printing error
AMMD: Absolute values of the maximum mean deviation per publication
IAE: Image acquisition error
SegC: Segmentation comparison error
lin: Linear
surf: Surface
nosim: No simulation
sim: Simulation
cad: Cadaver
pat: Patient
cal: Caliper
3Dcal: 3D-scan and virtual caliper
dis: Dissection
nodis: No dissection
DICOM: Digital imaging and communications in medicine
STL: Standard tessellation language
ISO: International organization for standardization
PRISMA: Preferred reporting items for systematic reviews and meta-analyses
RSNA: Radiological society of north america
MJP: Multi jet printing
PJP: Poly jet printing
DLP: Digital light processing
SLA: Stereolithography
LCD: Liquid crystal display
CLIP: Continuous liquid interface production
FDM: Fused deposition modeling
FFF: Fused filament fabrication
SLS: Selective laser sintering
SLM: Selective laser melting
CJP: Color jet printing
CAD: Computer aided design
CAM: Computer aided manufacturing
