# Supplementary figures and images for: Quality assurance of 3D-printed patient specific anatomical models: a systematic review
Source: 3D Print Med. 2024 Mar 27;10:9. doi: 10.1186/s41205-024-00210-5 (PMC10967057; doi:10.1186/s41205-024-00210-5)

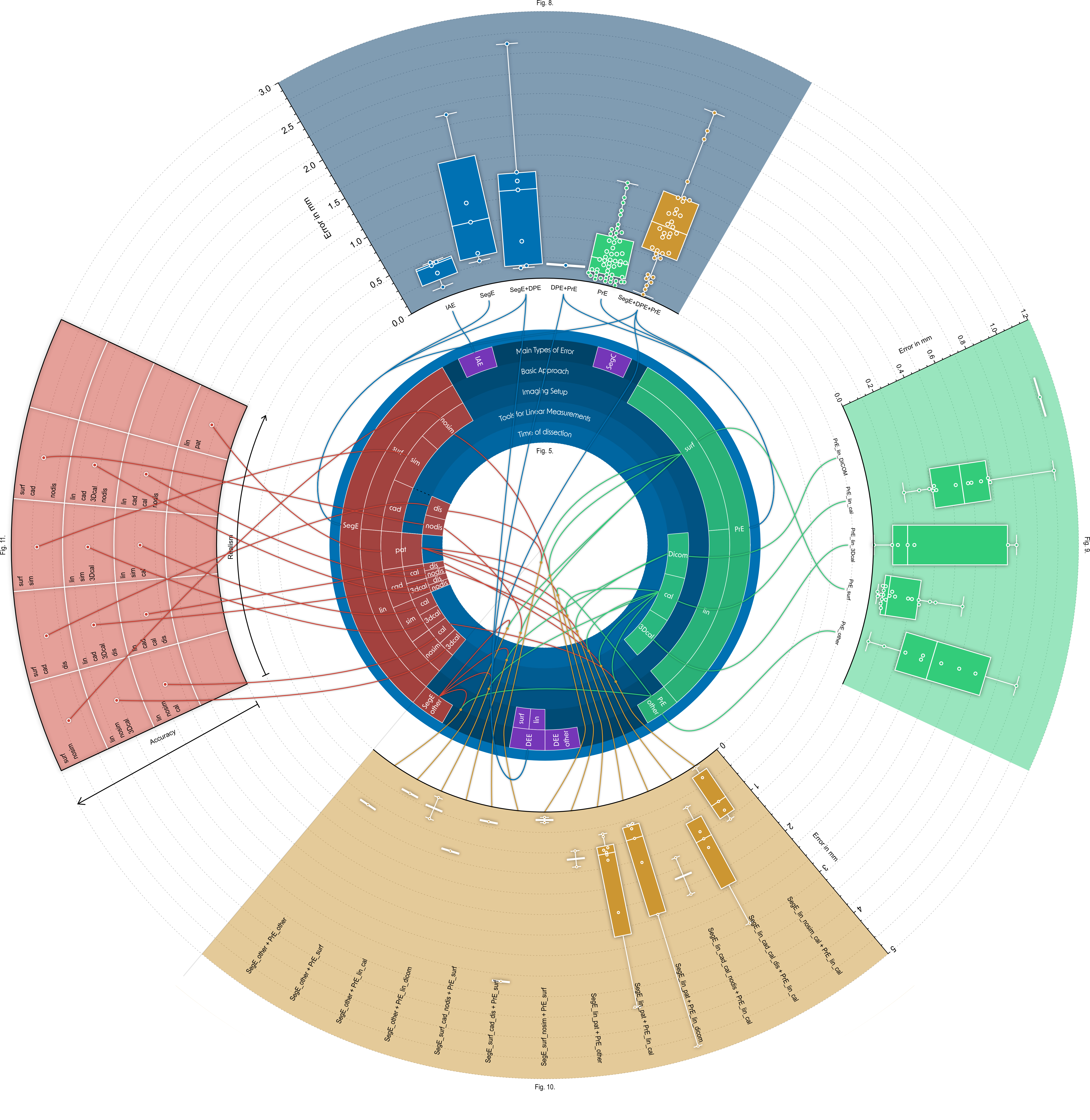

Supplement: Supplementary file 1 — Additional file 1:. High resolution version of the graphical abstract. [file 41205_2024_210_MOESM1_ESM.jpg]
